# Home Care Learning Model for Medical Students in Chile: A Mixed Methods Study

**DOI:** 10.1155/2014/631732

**Published:** 2014-01-16

**Authors:** Diego Garcia-Huidobro, Solange Rivera, Carolina Gonzalez

**Affiliations:** Department of Family Medicine, School of Medicine, Pontificia Universidad Catolica de Chile, Vicuña Mackenna 4686, Macul, Santiago, Chile

## Abstract

*Introduction*. The relevance of home care training is not questioned. However, there are no reported learning models to teach in this setting. *Aims*. To develop and evaluate a learning model to teach home care to medical students. *Methods. Stage 1: Learning Model Design.* Tutors teaching home care and a sample of medical students were invited to focus groups analyzed according to the grounded theory. Later, the researchers designed the learning model, which was approved by all participants. *Stage 2: Learning Assessment.* All students in their family medicine internship at Pontificia Universidad Catolica de Chile were invited to participate in a nonrandomized before-and-after pilot trial, assessing changes in their perception towards home care and satisfaction with the learning model. *Results*. *Stage 1:* Six tutors and eight students participated in the focus groups. The learning model includes activities before, during, and after the visits. *Stage 2:* 105 students (88.2%) participated. We observed improvement in all home care training domains (*P* ≤ 0.001) and a high satisfaction with the model. Students with previous home visit experiences and who participated with nurses and social workers reported more learning. *Conclusions*. We report an effective learning model to train medical students in home care. Limitations and recommendations for future studies are discussed.

## 1. Introduction

Home care is aimed at people that, due to their health condition, cannot access the healthcare system or for whom it is preferable to receive care in their domiciles. Because of the growing population of the elderly and the increasing life expectancy of people and the health system demands for early hospital discharge, home health services are increasing [1]. For all these reasons, the home is the preferred place for the health care of people with chronic disease, the terminally ill, and geriatric patients [2].

No matter what specialty medical students choose for their future, they will need to understand the context in which people live, the way that home services are delivered, and the unique complexity of home care. Therefore, it is necessary to incorporate teaching about home medical care into medical school curricula [1, 3–5].

The importance of this activity in the education of physicians concerns more than just clinical effectiveness [6]. Home care training has been described as an educational strategy that provides insights into the psychosocial, economic, and community determinants of health [1, 2, 7], all these elements being relevant to quality medical care.

The incorporation of home care as an educational activity requires preparation, implementation, and evaluation of the process [8, 9]. Even though some experiences have been published describing teaching at people's domiciles [10–12], there are no reports about learning models for home care, and few articles evaluate educational outcomes among students [13, 14]. Thus, the aims of this study were to design a learning model to teach home care to medical students and to evaluate their perceptions about learning with this model.

## 2. Methods

**Figure 1:**
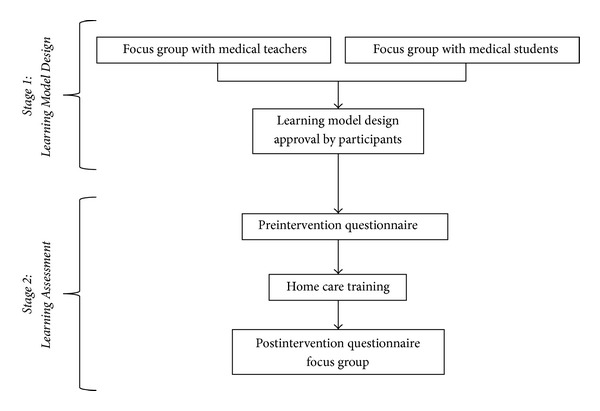
Study Design.

This study incorporated mixed methods integrating qualitative and quantitative methods using sequential exploratory mixed methods designs [15], in two stages, which are shown in Figure 1. First, we designed a learning model to teach home care for medical students using information collected in focus groups. Once the learning model was defined and implemented, we assessed the perceptions of students using a nonrandomized before-and-after pilot trial and a focus group. This combination of methods allowed the development of the learning model and then a comprehensive evaluation of its effects.

### 2.1. Setting

Medical education in Chile requires seven years of training after high school [16]. In the Pontificia Universidad Catolica de Chile, School of Medicine, last-year students have a mandatory seven-week rotation in Family Medicine, where students divide into three groups to work in different primary care clinics. As part of their internship, they are required to practice home visits during their rotation.

This study was conducted in three primary care clinics directed by the Department of Family Medicine. Each of these healthcare centers serves about 25,000 people and is located in underserved areas of Santiago, Chile. In these clinics, home visits are performed by family physicians, nurse practitioners, social workers, and medical assistants, directed to provide care for people.

### 2.2. Stage 1: Learning Model Design

We used a qualitative approach to collect the views of clinical tutors with experience conducting home visits with medical students (Figure 1) and the perceptions of a sample of students that had participated in home visits during their family medicine rotation.

Family physicians, nurses, and social workers that regularly practice home visits with medical students were invited to a focus group to share their teaching experiences, the methodology used to teach, and their perceptions about student learning with their personal approaches. To evaluate the perceptions of students towards home care, the different strategies used in their training, and their experience about learning in the patient's home, we invited a group of medical students to another focus group after they completed their rotation. Both focus groups were directed by a research assistant with experience in qualitative research. The sessions were audio recorded and transcribed. Then, these were codified and analyzed using Content Analysis [17], in NVivo 9 (Qualitative Solutions & Research Pty, 2002, Melbourne, Australia). Grounded theory [18] guided the development of the questions because the aim of these group interviews was to produce a learning model. Because we only conducted two focus groups, we used Content Analysis [17] to code the qualitative data, rather than the techniques proposed by Strauss and Corbin [18]. Steps included the development of an inductive coding schema and then coding the text. With the results of this analysis, the main researchers designed the learning model. To ensure that it considered the opinions of all participants, we invited all tutors to provide input on and to approve its final version.

### 2.3. Stage 2: Learning Assessment

To assess the learning of students who used this learning model, we used quantitative and qualitative approaches (Figure 1). First, we conducted a nonrandomized before-and-after pilot trial to evaluate changes in perception about learning. Later, at the end of the rotation, we conducted a new focus group with another sample of medical students. Questions were guided by a phenomenological approach [19] and their analysis used Content Analysis methods [17], as described above.

### 2.4. Procedures

During 2010, all students were invited to take part in the study at the beginning of their family medicine internship. If they agreed to participate, they were asked to read and sign an informed consent form. All procedures and consent forms were reviewed and approved by the Undergraduate Studies Committee and the Ethics Review Board of the School of Medicine of the Pontificia Universidad Catolica de Chile (approval number 09-224).

### 2.5. Home Care Training

During their seven-week rotation, students were required to participate in at least three home visits. All tutors agreed to use the designed learning model with their students. Because of the particular educational and healthcare characteristics of the clinics, the delivery of the learning model was different in the three participating healthcare centers. In the first clinic, students practiced home care exclusively with family physicians or family medicine residents; in the second, students went on home visits with a family physician, a nurse, and a social worker; and in the third clinic, the home care training was part of an interprofessional course, where medical students participated with nursing and psychology interns and practiced home care with a family physician, a nurse practitioner, and a social worker.

### 2.6. Outcomes

The primary outcome variable was the change in the total score of the medical student attitudes toward home care scale. Secondary outcomes were changes in the different domains of the scale and the students' perceptions about the learning model.

### 2.7. Instruments

Baseline assessment included a 26-item questionnaire divided into two sections: sociodemographics and a Spanish translation and adaptation of the medical student attitudes toward home care instrument [20]. This instrument includes 14 items assessing 4 domains: general attitudes, home care training, home-based therapies, and time and reimbursement (Cronbach's *α* 0.6–0.82 each domain). We modified and piloted the questionnaire before application. The final instrument consisted of 17 items divided into 3 domains: general attitudes (7 items), home care training (7 items), and home-based therapies (3 items). Each item was answered on a 1 to 7 scale. The fourth domain of the original scale (time and reimbursement) was excluded because patients did not pay for services provided. English translation of the questionnaire and internal reliability by domain are presented in the following list. The second assessment excluded sociodemographic questions.

 
*General Attitudes *(*α* = 0.672)
  (1) Home care is necessary for patients.
  (2) Respecting the privacy of patients is important in home care.
  (3) It is fulfilling to participate in the home care of bedbound patients.
  (4) Home care is useful for the management of certain conditions.
  (5) Team work is important for effective home care.
  (6) Adequate economic incentives could affect my decision of doing home care in the future.
  (7) Home visits are an efficient resource.
 
*Home-Based Therapies *(*α* = 0.606)
  (8) It is possible to perform a complete physical exam in the patients' home.
  (9) It is possible to use complementary exams in home visits.
  (10) It is possible to prescribe what is needed at the patients' homes.
 
*Home Care Training *(*α* = 0.801)
  (11) It is necessary to learn about home care during medical school.
  (12) In my medical training, I have learned about home care, and I feel capable of doing home visits alone.
  (13) During my medical training I have learned what a home care team does.
  (14) I am able to give indications at home for patients and their families.
  (15) I am able to differentiate patients who need and who do not need home care.
  (16) Through home care it is possible to learn about conditions that without home visits I would not learn.
  (17) Home visits allow assessing the environment of patients.

To assess the opinions of students about the learning model used in the second assessment, we included two questions evaluating if the model was adequate and useful for their learning. As well, we conducted a new focus group with students that completed their rotation in the three primary care clinics.

### 2.8. Statistical Analysis

Data analysis was performed with SPSS software v.16.1 (Chicago, IL, USA). We used paired *t*-tests for the analysis of the outcomes between the final and baseline measurements. Analysis of variance (ANOVA) was used to test differences of change in learning according to the clinic that students were assigned. To assess factors affecting the change in student's attitudes, we conducted multiple regression models studying the effect of age, gender, previous home visits, number of home visits during the rotation, and the effect of home visits with family physicians, nurses, and social workers. Multiple comparisons were taken into account using Bonferroni's adjustment. Resulting two-tailed *P* values of <0.05 were considered statistically significant.

## 3. Results

### 3.1. Stage 1: Learning Model Design

From a total of nine tutors who were invited, six participated in the first focus group: 4 family physicians, 1 nurse practitioner, and 1 social worker. All had experience of at least six months teaching home care to medical students. In the focus group of medical students, 8 students participated, representing the three different clinics.

The final home care learning model is presented in Table 1. The stages that should be considered when teaching home care are induction to the activity, patient selection, home visit preparation, activities during the home visit, discussion, and followup. Even though the tutor has final responsibility for clinical care and student teaching, the clinical and educational activities should be shared between the tutor and the students.

### 3.2. Stage 2: Learning Assessment

Of the complete cohort of students who completed their family medicine internship in 2010, 105 (88.2%) participated in the trial. A total of 60 students were male (57.1%), with 24.7 ± 1.2 years. Most students had performed home visits previously in their training (95, 90.5%). Students were excluded if they missed one of the two assessments (12, 10.1%) or if they refused to participate (2, 1.6%).

Perceptions of all students before and after participating in home care teaching are summarized in Table 2. We observed a significant change in the total score of the instrument (10.8 pts, *P* < 0.001). The most important change between the final and baseline assessments was observed in the home care training dimension, which included questions as to whether providing home care is gratifying, useful, or necessary for patients and whether respect for patients and team work is important in the care of homebound patients. The general attitudes and home-based therapies dimensions had very high baseline scores and reported the lower change between the two assessments.

Multiple regression analysis showed that the factors associated with improvement in the total score were number of home visits previous to the internship, home visits with a social worker, and home visits with a nurse practitioner (adjusted R2 0.587, *P* = 0.003). All these elements were positively associated with the independent variable in the final model.

As observed in Table 3, a training program that taught home care with students from other health disciplines, as well with interprofessional tutors (family physician, nurse practitioner, and social worker), produced greater improvement in the home care training domain than the clinic without interdisciplinary students (*P* = 0.006) and interdisciplinary tutors (*P* = 0.101). These differences persisted after modeling the change in perceptions by gender, age, number of home visits previously, and number of home visits during the internship; however these were not observed in the total score or the other domains studied.

Students scored 6.0 ± 1.2 (mean ± SD) in response to the question of whether the learning model was adequate and scored 6.0 ± 1.3 (mean ± SD) in response to whether the model contributed to their learning, both scores on 1 to 7 ranges.

Nine students from the 3 clinics participated in the postintervention focus group. Similarly to students in the first focus group, they expressed that the home visit experiences contributed to teamwork learning, to having a systemic orientation to people, and to developing comprehensive clinical health plans. However, their perceptions differed regarding the learning model used and the learning process. After conducting home visits using the learning model detailed above, they perceived learning regarding prioritizing health problems during the home visits, developing communication skills with patients and family members, to limit the patient-physician relationship. Students considered this learning model appropriate and that it provided a clinical framework for conducting future home visits. Examples of students' opinions about home care training, the teaching strategy, and suggestions for improvement (second focus group): are summarized in the following list.

 
*Home Care Training*
  - “The main use of home visits is to assess patients in their own environment and consider their reality in the context of their management. This contributes to a comprehensive view of patients that complements diagnosis and prescribed treatments.”
  - “When you practice with people from other disciplines, you learn their language: what they see, what they ask, what they say. You learn from them more than you learn from your own classmates [medical students] because they are trained so differently from us.”
  - “Home care allows us to have closer medical relationships with patients and family members because we know their real environment.”
  - “In people's homes you become more human, you empathize better with their needs.”
 
*Learning Model*
  - “I give a 7 [maximum score] to the home care training. The tutors were always very interested in teaching us.”
  - “It's very important that the home visits have a clear objective. If so, it's easy, because you know clearly what you need to do.”
  - “The framework presented helped us to learn with the patients we saw, not just going to their homes, doing the work, and writing in the medical record. We learned before and after going to people's homes.”
 
*Suggestions for Improvement*
  - “We should follow-up with the patients and families visited during our rotation to see what the effects of our interventions were.”
  - “Time set aside specifically for updating the patients' medical records will help us to avoid leaving out important information.”

## 4. Discussion

In this paper, we report the design and evaluation of a learning model to teach home care to medical students in primary care. Using this model, we observed an improvement in the students' perceptions towards home care services from quantitative and qualitative perspectives.

Even though multiple home care training programs have been reported [13, 21–23], up to the best of our knowledge this is the first paper that describes the development of a learning model to teach home care. Considering the opinions of a multidisciplinary team of teachers and medical students, we recommend that the learning model includes before, during, and after home visit activities, in order to facilitate student's learning (Table 1). Particularly important is to provide a different opportunity for the case analysis, before giving the final indications to the patients or family members. As well, we consider that educational activities should be distributed between students and tutors to facilitate the active learning of interns. More research is needed to learn about potential modifications of this model.

Students were satisfied when learning with the model presented above; however the qualitative evaluation was limited and future studies should include more focus groups to ensure the trustworthiness of the reported findings. Suggestions for improvement include structuring checkups of the patients visited during the student's rotation and providing time to update the electronic records of patients. These recommendations should guide modifications of the reported model.

Although we designed this learning model for medical students, this approach can be implemented to teach home care to family medicine residents or students of other disciplines. Residents also require supervision and guidance in their learning of home care, since it is uncommon that this type of care is taught in medical school [1–4]. This model was successfully applied in an interdisciplinary course with medical, nursing, and psychology students [24]; however, more research is needed to identify the key elements of home care teaching strategies that need to be included in educating other professionals.

In this study we observed a significant improvement in the perceptions of students towards home care, home care training, and self-perception in terms of providing care in the patient's house using this learning model. These findings are consistent with those of other studies that have shown a positive effect of educational programs on student attitudes toward home care [13, 22]. Flaherty and colleagues observed an improvement in general attitudes toward home-based therapies and home care training in an educational program directed at third- and fourth-year students in 5 medical schools [13]. As in this study, they observed that the greatest change from prerotation to postrotation assessments was in the home care training domain. Also, Neale and colleagues evaluated the perception of family medicine residents at the end of a home care program [22]. After a three-month rotation, residents considered that home visits were an important part of residency training and observed improved knowledge of geriatric medicine, patient compliance issues, patient functional status assessment, and community service use.

In addition, we observed that using this learning model with participation of students and tutors from other disciplines was associated with a greater improvement in students' perceptions towards home care (Table 3). Even though we were not able to adjust to all possible confounding variables (such as medical school grades prior to the family medicine rotation), this result supports the model that interprofessional educational training provides greater learning opportunities than traditional one-discipline preparation, producing better outcomes in students [24, 25]. Because we did not observe different scores in the students supervised by interprofessional tutors compared to students taught exclusively by medical tutors, these effect should be attributed to the interprofessional student learning experience. However, more research is needed to understand which elements of educational strategies are associated with increased learning in home care training and to further corroborate this finding.

This paper has several limitations that are important to address. First, to evaluate the impact of the learning model, we designed a nonrandomized before-and-after pilot trial, without a control group of students. Because we considered that all students might benefit from this educational intervention, we did not want to exclude students from this learning opportunity and have them allocated to a control group. To mitigate the effect of an absent control group, we assessed home care domains that were unlikely to be affected by other experiences during their family medicine rotation. However, randomized trials are the most useful way to prove causality and to control bias and therefore should be employed in future studies [26].

Second, we assessed perceptions and attitudes towards home care, second stages in educational outcomes [27]. Further research should include the perceptions of teachers and patients and assess higher educational impacts, such as skills and behavior, to finally affect service delivery and patient care. The costs of these educational interventions should also be evaluated, considering the possible benefits of interprofessional tutoring.

## 5. Conclusion

In this paper we report the development of a learning model to teach home care to medical students, with a positive assessment according to the participants' perceptions. These results support the use of home care training programs for medical students to improve their attitudes toward home care and to increase the efficacy of the care they provide in the patient's domicile. Because of the scarcity of educational reports on home care teaching, further research is needed to draw conclusions about home care training in medical education.

## Figures and Tables

**Table 1:** Home care learning model.

Stage	Description	Responsible
Induction	Tutors describe the activities during the home visit and explain the learning model. We encourage the use of handouts.	Tutor

Patient selection	Tutors tell the students about the case for the home visit days before, and the students check the medical record of the patient. One student is responsible for presenting the clinical history.	Tutor

Home visit preparation	One student presents a summary of the clinical history of the patient or family that will be visited. Students define the following:	Students
	(i) problems of the patient or family,	
	(ii) objectives for the home visit,	
	(iii) materials needed for the visit (sphygmomanometer, prescription or referral forms, wound healing materials, clinical scales for patients or family members, etc.),	
	(iv) student roles during the visit.	

Home visit	Tutor presents his/her team, ensuring that the students assume their roles. At the end the tutor explains to the patient or family members that a comprehensive health plan will be discussed, and that a student will make a telephone call or schedule an appointment to explain the treatment alternatives for his/her relative (in the case that the patient does not need an urgent referral to the emergency room).	Tutor leads and students assume their roles

Discussion of the visit	Biomedical and psychosocial problems are reassessed, including supportive factors of patients and their families. A genogram is completed according to the needs of the patients. For each problem, possible solutions are discussed according to their effectiveness and likelihood to be implemented at home. Followup is scheduled. According to the visit, clinical questions are identified and distributed to students to be answered and discussed during the next session.	Tutor facilitates the discussion

Followup	Followup with all patients. This activity is registered in the record of the patient. Students answer the clinical questions from previous visits and review the unknown contents and share in their learning.	Tutor facilitates group learning

**Table 2:** Perception of students before and after participating in home care teaching program (mean ± SD).

Domain (score)	Before	After	*P* value
General attitudes (7–49)	43.2 ± 4.8	45.3 ± 4.5	<0.001
Home care training (7–49)	34.3 ± 6.6	41.9 ± 4.8	<0.001
Home-based therapies (3–21)	16.4 ± 2.9	17.4 ± 2.4	0.001

Total score (17–119)	93.8 ± 14.3	104.6 ± 16.5	<0.001

**Table 3:** Change in the perception of students according to the home care training setting (mean difference ± SD).

Domain	Interprofessional students, interprofessional tutors (*n* = 40)	Medical students, interprofessional tutors (*n* = 25)	Medical students, medical tutors (*n* = 40)	Unadjusted *P* value	Adjusted *P* value
General attitudes	1.8 ± 4.2	2.3 ± 4.7	2.3 ± 4.6	0.828	1
Home care training	9.8 ± 6.5	5.2 ± 4.5	7.0 ± 5.7	0.006	0.021
Home-based therapies	0.9 ± 3.4	0.2 ± 2.8	1.6 ± 2.6	0.197	0.494

Total score	12.5 ± 10.8	7.9 ± 8.0	10.8 ± 8.2	0.175	0.383
